# The antimalarial resistome – finding new drug targets and their modes of action

**DOI:** 10.1016/j.mib.2020.06.004

**Published:** 2020-10

**Authors:** Krypton Carolino, Elizabeth A Winzeler

**Affiliations:** 1 Department of Biological Sciences, University of California, San Diego, La Jolla, CA 92093, United States; 2 Department of Pediatrics, University of California, San Diego, School of Medicine, La Jolla, CA 92093, United States

## Abstract

To this day, malaria remains a global burden, affecting millions of people, especially those in sub-Saharan Africa and Asia. The rise of drug resistance to current antimalarial treatments, including artemisinin-based combination therapies, has made discovering new small molecule compounds with novel modes of action an urgent matter. The concerted effort to construct enormous compound libraries and develop high-throughput phenotypic screening assays to find compounds effective at specifically clearing malaria-causing *Plasmodium* parasites at any stage of the life cycle has provided many antimalarial prospects, but does not indicate their target or mode of action. Here, we review recent advances in antimalarial drug discovery efforts, focusing on the following ‘omics’ approaches in mode of action studies: IVIEWGA, CETSA, metabolomic profiling.

## Highlights

• *In-vitro* evolution and whole genome sequencing elucidates gene–drug interactions.
• Cellular thermal shift assay and mass spectrometry directly reveals small molecule compound–protein interactions.
• Metabolomic profiling complements target identification with broad mode of action.

**Current Opinion in Microbiology** 2020, **57**:49–55

This review comes from a themed issue on **Antimicrobials**

Edited by **Iruka N Okeke** and **Audrey R Odom John**

For a complete overview see the Issue and the Editorial

Available online 15th July 2020

**https://doi.org/10.1016/j.mib.2020.06.004**

1369-5274/© 2020 The Authors. Published by Elsevier Ltd. This is an open access article under the CC BY license (http://creativecommons.org/licenses/by/4.0/).

## Introduction

Tropical diseases remain a prevalent source of morbidity and mortality globally. Malaria, arguably the most widespread parasitic infection in humans, is mainly caused by protozoan parasite species *Plasmodium falciparum* and *P. vivax*. Humans contract malaria through the bite of an infected female mosquito, releasing *Plasmodium* sporozoites that first travel to the host liver, where they mature into schizonts. Schizonts rupture and release merozoites back into the bloodstream, where they invade red blood cells. Merozoites mature into schizonts that in turn generate more merozoites that will invade other red blood cells when they burst out of their current cells. Aside from the asexual blood stage, there is the sexual blood stage in which merozoites mature into gametocytes that are taken up by mosquitoes taking a blood meal, where they develop into sporozoites [1]. These sporozoites are then transmitted to the next human by the next mosquito blood feed, thus continuing the lifecycle.

In 2018, there were an estimated 228 million cases of malaria worldwide (an increase of nine million cases over 2017), 405 000 of which resulted in death [2]. Malaria remains a significant burden despite preventative measures (currently chemoprevention, insecticides, and insecticide-treated bed nets). Parasite resistance to current antimalarial drugs (including first-line artemisinin-based combination therapies, ACTs), as well as mosquito resistance to insecticides (whether behavioral, physiological, or biochemical [3]), has threatened malaria eradication efforts. Developing innovative vector control measures like CRISPR-based gene drives [4], as well as novel treatment/prevention strategies such as monoclonal antibodies [5], vaccines [6], or antimalarial drugs with novel modes of action are needed more than ever.

One approach to developing new drugs involves identifying small molecules with antiparasitic activity using phenotypic screening — testing compound directly against living parasites. The concerted efforts by many labs to design high-throughput phenotypic screening methods for the entire *Plasmodium* lifecycle and assemble chemical libraries have led to the discovery of many compound candidates effective in parasite clearance [7]. This approach has led to new clinical candidates such as KAE609 (cipargamin) and KAF156 (ganaplacide), which are currently in phase IIb clinical trials. A recent screen of over 500 000 compounds yielded 681 candidates with submicromolar efficacy at the liver stage [8^••^]. Another screen of almost 70 000 compounds yielded 17 candidates with transmission blocking activity [9]. A limitation to these screens is that they do not specify targets. Thus, many candidate hits are being re-screened in specific assays to determine putative mechanism of action. The Tres Cantos Antimalarial Compound Set was re-screened in assays specific for transmission blocking: TCMDC-135461 and TCMDC-137173 target cytochrome bc1; TCMDC-134114 targets dihydrofolate reductase, and TCMDC-125849 targets Pfs25 [10]. It was also re-screened in assays specific for respiratory inhibition [11] and proteasome inhibition [12].

Additionally, as the rate of discovery of new antimalarial chemotypes decreases, many are reconsidering target-specific screens, like a recent biochemical screen designed to identify inhibitors of lysyl-tRNA synthetase [13]. Such screens can be less expensive to run, do not require specialized access to parasites, and could theoretically give leads with improved potency and selectivity, especially when combined with knowledge of the target’s structure. Two promising compounds in clinical trials, DSM265 and P218 were found to target DHODH and DHFR, respectively, using this method. The current bottleneck now lies in identifying high quality targets, such as lysyl-tRNA synthetase. A bioinformatics approach can be taken to predict alternative binding sites in *Plasmodium* aminoacyl tRNA synthetases that have low homology to those in humans, thus making them potential druggable targets [14]. Here, the computational approach is expanded as we discuss current methods for discovering antimalarial drug targets with novel modes of action through ‘omics’ approaches – genomics, proteomics, metabolomics.

## *In-vitro* evolution and whole genome analysis unveils novel target genes

**Figure 1:**
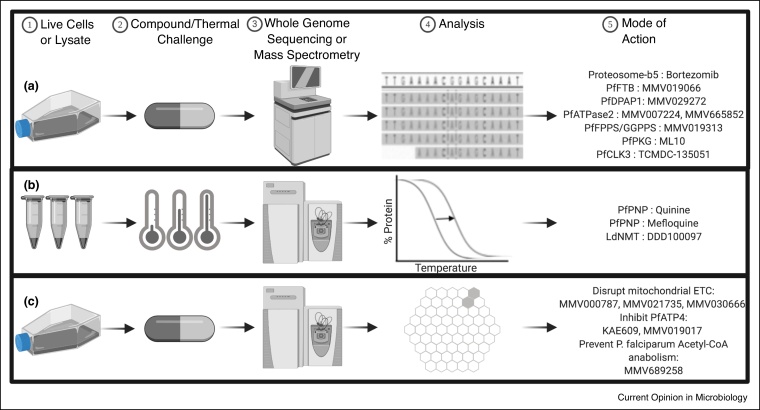
Overview of IVIEWGA, CETSA, and metabolomic profiling processes. **(a)** IVIEWGA comprises exposing drug-naive P. falciparum parasites to sublethal doses of a compound until resistance is observed via an upwards shift in IC50 concentration. Parasites are prepared for whole genome sequencing, followed by call variant comparisons to the parental strain. **(b)** CETSA consists of mixing compound, or vehicle, with *P. falciparum* parasite lysates and exposing mixtures to a range of temperatures, from 37C to 73C. Samples are prepared for LC/MS–MS, yielding melt curves for each protein. **(c)** In metabolomic profiling, *P. falciparum* parasites are treated with compound, or vehicle, followed by metabolite extraction. Samples are prepared for LC/MS–MS, generating a metabolic fingerprint (metaprint) for each compound. Each individual hexagon represents a metabolic pathway; the shading determines fold change.

A promising method for discovering new targets involves *in-vitro* evolution and whole genome analysis (IVIEWGA). This method comprises exposing *P. falciparum* parasites to sublethal doses of a compound until an upward shift in IC_50_ is observed in the culture, indicative of resistant parasites. Sequenced genomes of the resistant parasites are compared to those of the drug-naive parent to reveal genetic changes such as single nucleotide polymorphisms (SNPs) and copy number variants (CNVs). These genomic changes can point to genes encoding drug targets, but can also point to genes encoding multidrug resistance, such as the *P. falciparum* multidrug resistance 2, PfMDR2 [15] or improving the fitness of a drug-selected line. They may even be just random background mutations. Distinguishing whether a gene encodes a target or a resistance mechanism can be challenging, especially if the mutation is in a currently uncharacterized genes, as was the case with the *P. falciparum* Niemann-Pick Type C1-Related protein, PfNCR1, now deemed important for parasite plasma membrane composition and digestive vacuole biogenesis [16]. Recently, this method has uncovered or reconfirmed additional new antimalarial drug targets, notably: the proteasome [17], thymidylate synthase, farnesyltransferase (PfFTB), dipeptidyl aminopeptidase 1 (PfDPAP1), aminophospholipid-transporting P-type ATPase (PfATPase2) [18^••^], bifunctional farnesyl/geranylgeranyl diphosphate synthase (PfFPPS/GGPPS) [19], cGMP-dependent protein kinase (PfPKG) [20], and cyclin-dependent-like kinase 3 [21] (Figure 1a). Other drug targets discovered through IVIEWGA are summarized in this review [22]. In a few cases, resistant lines have been created but it is not clear which mutation gives resistance, potentially because resistance is conferred by a mutation in an AT-rich intergenic region. Whole genome sequencing is advancing to allow deeper coverage and longer reads (that can help repetitive intergenic tracts) and bioinformatics pipelines are improving to better detect SNPs and CNVs. Machine-learning may be used to predict which of these are likely to confer resistance without laborious manual confirmation via CRISPR-Cas9 genome editing or inducible TetR-aptamer systems [23].

‘Irresistible’ compounds are those that have failed to generate resistant *P. falciparum* lines. They are interesting because if developed into drugs, they may be less likely to fail early from acquired drug resistance. For these compounds, IVIEWGA could be attempted in closely related organisms, like *Toxoplasma gondii* [24]. Other methods are required to study these irresistibles, such as proteomics and metabolomics discussed below. Furthermore, as molecular biology techniques advance, generation of *Plasmodium* overexpression libraries will be feasible, allowing for more efficient target identification via IVIEWGA.

## Affinity-based and protein-stability assays can directly identify and validate target genes

**Figure 2:**
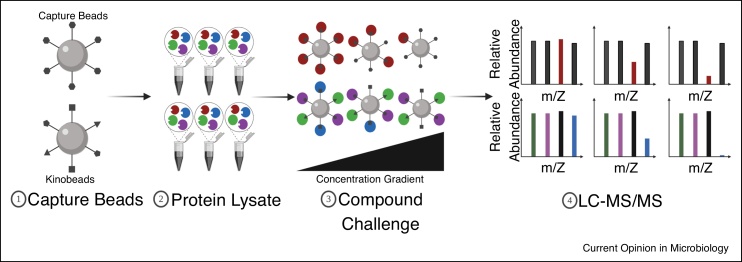
Overview of chemoproteomics pull-down competition approach. Capture beads are prepared by covalently immobilizing an analog compound (top) to Sepharose beads; should ATP-competitive kinase inhibitors be bound, one would generate Kinobeads (bottom). These beads are added to *P. falciparum* lysates, with increasing amounts of compound of interest. As compound of interest increases, it will compete with ligands attached to the beads for the target protein. Upon analysis of proteins bound to the beads via LC/MS–MS, there should be a decrease in the abundance of the target protein, as seen between MMV66685 snd PfPI4K when MMV390048 is added [28].

Proteomics has been used to study *Plasmodium* protein expression and post-translational modifications, integral in finding regulatory mechanisms that can be exploited for antimalarial drug discovery [25]. It can also be utilized to remedy limitations of IVIEWGA through approaches like affinity-based or protein-stability assays [26]. In the popular pull-down method, a tag bound covalently to a compound of interest attaches to interacting protein targets, which can then be isolated from other proteins via centrifugation, magnetic separation, or click chemistry, depending on the tag used. Interacting proteins, now enriched, are prepared for liquid chromatography followed by mass spectrometry (LC/MS—MS) so they can be identified. Recently, an alkyne tag on a photoactivatable group-diaminoquinazoline compound probe was used to bind its molecular target via UV-activated click chemistry; LC/MS—MS identified 104 candidate target genes [27]. Because pull-downs can yield false positives through non-specific binding, they should also be used in competition assays. *P. falciparum* cell extracts are incubated with heavy Sepharose beads having an analog compound, like MMV666845, or adenosine triphosphate (ATP)-competitive kinase inhibitors (Kinobeads) attached; as the compound of interest, like MMV390048, increases, the target protein should bind less to the beads (Figure 2). And by cross-referencing pull-down data with that from IVIEWGA, candidates can be narrowed down to one target, as seen with the MMV390048-phosphatidylinositol 4-kinase (PfPI4K) interaction [28]. Moreover, approaches like protein-protein immunoprecipitation studies can be used in elucidating mechanisms of action, as seen with *P. falciparum* histone deacetylase 1, PfHDAC1, interacting with 26 complex proteins [29]. Though, a limitation to pull-downs is synthesis of these tag-bound small molecules and subsequent testing for efficacy, as the tag may neutralize affinity or decrease potency, requiring structure-activity relationship optimization.

**Figure 3:**
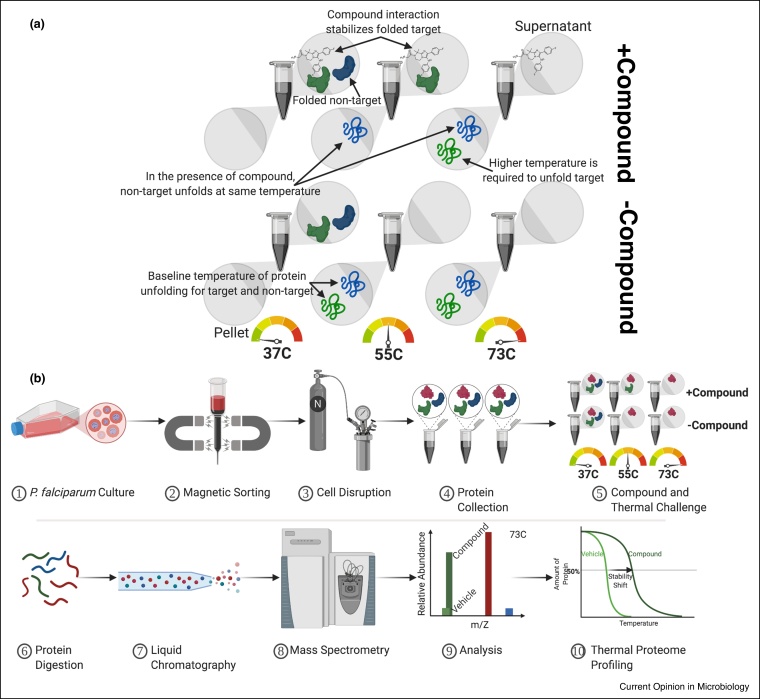
Schematic of the cellular thermal shift assay. **(a)** Late-stage *P. falciparum* parasites are concentrated via magnetic isolation to minimize protein masking by hemoglobin. Protein lysates generated after nitrogen cavitation and cell disruption are subject to treatment with compound, or control, followed by exposure to a range of temperatures to determine protein stabilities. Sample supernatants are digested and TMT-labeled for LC/MS–MS processing. Thermal proteome profiling comprises generating stability shift curves for each protein detected, looking for increased temperature to denature 50% of a protein. **(b)** Presence of the compound during compound and thermal challenge stabilizes the target protein, requiring a higher temperature to denature 50% of that protein. Stabilization keeps more target protein in the supernatant relative to the pellet.

To circumvent compound alteration, one can utilize the emerging label-free cellular thermal shift assay (CETSA), or thermal proteome profiling (TPP), another method that does not require previous knowledge on compound mode of action. This experiment relies on biophysical properties of proteins, while in their native environments even, to unravel interactions with small molecules. When cells are treated with a compound of interest, the compound binds to its protein target. When challenged with heat, unbound proteins denature and precipitate out of solution, whereas compound-bound proteins are stabilized and thus require higher temperature to precipitate, relative to the vehicle control. A protein, identified via LC/MS-MS, with an increased temperature shift suggests that it is binding to the compound and possibly the direct target [30] (Figure 3). This method has recently been developed for parasites, like *Leishmania donovani* [31] and *P. falciparum*. Quinine and mefloquine were found to bind to purine nucleoside phosphorylase, PfPNP [32^••^] (Figure 1b). The drawback of this assay is that it has currently been developed to identify soluble proteins only, and detection is not always possible when endogenous protein levels are low [33]. If compounds have an alternative mechanism of action responsible for their activity, such as binding hemoglobin, RNA, or lipids, protein-binding events may be real but obscure the true mechanism of action. Again, specificity of drug target interactions, along with downstream stability fluctuations, can yield false positives. Thus, this method still requires additional validation. There is promise for high-throughput CETSA that utilizes NanoLuciferase reporter and AlphaLISA assay to identify compounds directly targeting a specific protein [34]. Also, there are other stability-based proteomics techniques waiting to be harnessed in malaria research [35].

## Metabolomic profiling and transcriptomics narrows down the mode of action

Metabolomic profiling comprises treating *P. falciparum* parasites with a compound of interest, extracting and analyzing the metabolites via LC–MS, and comparing the metabolomics fingerprint, or metaprint, to that of untreated parasites [36]. This method may not provide the exact target, but it narrows down the mode of action, valuable for target validation or characterization. One caveat is that metabolite changes could be attributed to non-specific or non-viable phenotypes, but this can be overcome with testing at various concentrations or over a period of time [36]. Recently, MMV000787, MMV021735, and MMV030666 showed a profile akin to DSM265 and atovaquone, which disrupt the mitochondrial electron transport chain via inhibition of dihydroorotate dehydrogenase and cytochrome bc1, respectively, observed through an increase in pyrimidine precursors dihydroorotate and *N*-carbamoyl-l-aspartate. Additionally, KAE609 and MMV019017 showed a profile of decreasing nucleoside di-phosphate and tri-phosphate levels, a signature of inhibiting Na+-dependent/H+-dependent ATPase PfATP4 [37]. Another study found that pantothenamides, like MMV689258, sequesters coenzyme A to block acetyl-CoA anabolism, observed through CoA analog buildup [38^••^] (Figure 1c). A limitation is the possibility that compounds do not act through metabolic pathway disruption. And as with other big data sets, metabolomics studies are susceptible to confounding variables, such as host contamination and the normalization approach, which need to be addressed [39].

The pre-erythrocytic stage remains of particular interest for antimalarial drug discovery because, with fewer parasites, resistance is less likely to emerge at this stage. Recently, genome-wide RNA-seq performed for *Plasmodium berghei* exoerythrocytic stages provided insight into the metabolic networks of these parasites during development in the liver [40]. When combining these findings with those from a whole lifecycle phenotypic screen of barcoded knockout mutant *P. berghei* parasites, a liver-stage metabolic model was generated, which indicated that type II fatty acid synthesis and elongation, tricarboxylic acid, amino sugar, heme, lipoate, and shikimate metabolism to be essential for parasite proliferation and thus potential drug targets in the liver stage [41]. Moreover, transmission-blocking agents are also of interest to prevent new malaria cases. Combining RNA-seq and proteomics in characterizing oocyst and salivary gland sporozoites of *Plasmodium yoelii* and *P. falciparum* provides insight in the mechanism of parasite transmission, which can be targeted for novel action drug discovery [42].

## Conclusion

Taking an omics approach is the future of novel antimalarial drug discovery [43]. Millions of compounds have been screened for antimalarial acitivity. The bottleneck to drug discovery occurs in drug target identification and mode of action. The most used, genomic method of IVIEWGA generates lists of target genes that are muddied by background noise. Therefore, complementary assays are required, those investigating the *Plasmodium* transcriptome, proteome, and metabolome [44]. Involving CETSA in target validation, the potential to develop drugs with novel mechanisms within the near future is more feasible. Metabolomic profiling further helps prioritize compounds for lead optimization. Compounds targeting the food vacuole, metabolic processes, mitochondria, and transport pathways have been discovered [45]. Since not every compound can go to clinical trials, mechanistic and pharmacokinetic studies can help identify and prioritize compounds [46].

Though the *P. falciparum* genome has been fully sequenced, many genes still lack functional predictions, in part because of the dearth of malaria researchers. With the release of Malaria Cell Atlas [47^••^], there is a now an initial, and growing source for gene function and mechanism information for the parasite across its complex lifecycle, rather than just focusing on the asexual blood stage. This atlas can serve as a reference for understanding functions of genes targeted by various compounds throughout the parasite lifecycle. These-omics approaches should be further developed for *P. vivax*, the other species responsible for a majority of malaria cases worldwide, and one with a unique hypnozoite stage of development [48]. Additionally, with many natural products shown to have antimalarial activity [49], it would be beneficial to identify their target and mechanism of action through these – omics approaches. Lastly, not only are antimalarial drugs with novel modes of action needed but also vaccines, or novel vector control methods to reach malaria eradication [50].

## Conflict of interest statement

Nothing declared.

## References and recommended reading

Papers of particular interest, published within the period of review, have been highlighted as:

•• of outstanding interest

## CRediT authorship contribution statement

**Krypton Carolino:** Conceptualization, Investigation, Data curation, Visualization, Funding acquisition, Writing - original draft, Writing - review & editing. **Elizabeth A Winzeler:** Data curation, Resources, Supervision, Funding acquisition, Writing - review & editing.
